# Supplementary material for: Understanding Immune Dynamics in Liver Transplant Through Mathematical Modeling
Source: Bull Math Biol. 2025 Jul 19;87(8):112. doi: 10.1007/s11538-025-01480-8 (PMC12276165; doi:10.1007/s11538-025-01480-8)
Supplement: Supplementary file 1 — Supplementary file1 (DOCX 346 KB) [file 11538_2025_1480_MOESM1_ESM.docx]

Article Title: Understanding Immune Dynamics in Liver Transplant Through Mathematical Modeling

Journal Name: Bulletin of Mathematical Biology

Author Names: Julia Bruner, Kyle Adams, Skylar Grey, Mahya Aghaee, Sergio Duarte, Ali Zarrinpar, Helen Moore

Corresponding Authors’ Email Addresses: [juliabruner@ufl.edu](mailto:juliabruner@ufl.edu), [helen.moore@medicine.ufl.edu](mailto:helen.moore@medicine.ufl.edu)

| **Supplementary Table 1: Total Sensitivity Indices, S_T_, with Different Numbers of Base Samples** | | | | |
| --- | --- | --- | --- | --- |
| **Parameter** | **Indices from 175,000 Samples** | **Indices from 150,000 Samples** | **Indices from 125,000 Samples** | **Indices from 100,000 Samples** |
| [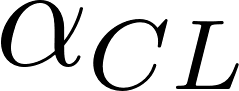](https://www.codecogs.com/eqnedit.php?latex=%5Calpha_%7BCL%7D#0) | 0.347559152442801 | 0.347729699245278 | 0.347390993437873 | 0.347091185038593 |
| [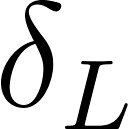](https://www.codecogs.com/eqnedit.php?latex=%5Cdelta_L#0) | 0.347095708804508 | 0.347173369671217 | 0.347340645230406 | 0.346866546371983 |
| [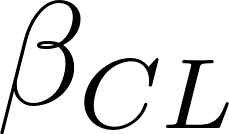](https://www.codecogs.com/eqnedit.php?latex=%5Cbeta_%7BCL%7D#0) | 0.0622289305920515 | 0.0622185062990897 | 0.0623341255775582 | 0.0624034615919833 |
| [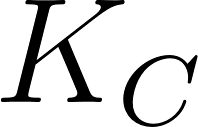](https://www.codecogs.com/eqnedit.php?latex=K_C#0) | 0.0613353561245457 | 0.0614622092467039 | 0.0613955209379824 | 0.0611237663834724 |
| [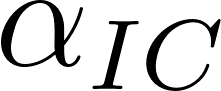](https://www.codecogs.com/eqnedit.php?latex=%5Calpha_%7BIC%7D#0) | 0.0542233341511756 | 0.0545498713388201 | 0.0544719704033234 | 0.0551113562878893 |
| [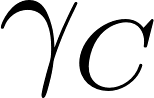](https://www.codecogs.com/eqnedit.php?latex=%5Cgamma_C#0) | 0.0535616768046202 | 0.0532846991866855 | 0.0530811158633552 | 0.0533025124370457 |
| [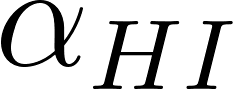](https://www.codecogs.com/eqnedit.php?latex=%5Calpha_%7BHI%7D#0) | 0.0455036460628587 | 0.0448192149190912 | 0.0448873801378925 | 0.0448573974616393 |
| [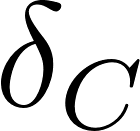](https://www.codecogs.com/eqnedit.php?latex=%5Cdelta_C#0) | 0.0404086679210438 | 0.0405828979298113 | 0.0407228321627269 | 0.0410115147201916 |
| [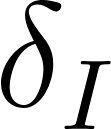](https://www.codecogs.com/eqnedit.php?latex=%5Cdelta_I#0) | 0.034695122009486 | 0.0347175879508261 | 0.0346887868741547 | 0.0342083422711859 |
| [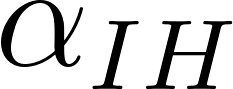](https://www.codecogs.com/eqnedit.php?latex=%5Calpha_%7BIH%7D#0) | 0.031257447526204 | 0.0314963858014313 | 0.031674671469232 | 0.030748920630857 |
| [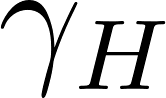](https://www.codecogs.com/eqnedit.php?latex=%5Cgamma_H#0) | 0.0309166369358803 | 0.0306933179385967 | 0.0304766808310024 | 0.0296002919736201 |
| [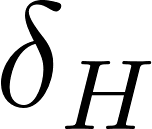](https://www.codecogs.com/eqnedit.php?latex=%5Cdelta_H#0) | 0.0268674847020804 | 0.0268108040590406 | 0.0265733810545274 | 0.0262743075330643 |
| [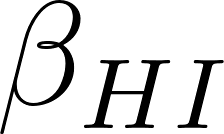](https://www.codecogs.com/eqnedit.php?latex=%5Cbeta_%7BHI%7D#0) | 0.01480383522852 | 0.0147054933998825 | 0.0147159640269229 | 0.0144093550722028 |
| [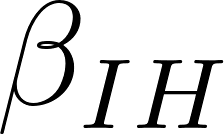](https://www.codecogs.com/eqnedit.php?latex=%5Cbeta_%7BIH%7D#0) | 0.0144515077526366 | 0.01475844529688 | 0.0148648006619356 | 0.0144050858832162 |
| [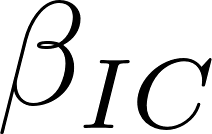](https://www.codecogs.com/eqnedit.php?latex=%5Cbeta_%7BIC%7D#0) | 0.0120771175426854 | 0.0118264120605982 | 0.0117141260334143 | 0.0119591377270486 |
| [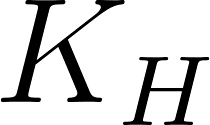](https://www.codecogs.com/eqnedit.php?latex=K_H#0) | 0.00746101341864828 | 0.00739841464905094 | 0.00730804504374788 | 0.00711355862452181 |
| [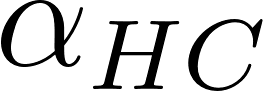](https://www.codecogs.com/eqnedit.php?latex=%5Calpha_%7BHC%7D#0) | 1.93818556936963e-05 | 2.04790818671106e-05 | 1.09073540645157e-05 | -6.39385110612942e-06 |
| [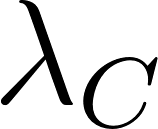](https://www.codecogs.com/eqnedit.php?latex=%5Clambda_C#0) | 1.08796465315924e-05 | 6.15037180129074e-06 | 6.94878407032467e-06 | 5.55418968566871e-06 |
| [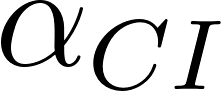](https://www.codecogs.com/eqnedit.php?latex=%5Calpha_%7BCI%7D#0) | 9.0269534605571e-06 | 7.47634488420676e-06 | 6.46943365823947e-06 | 4.91540131520099e-06 |
| [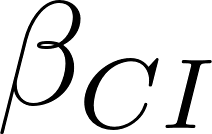](https://www.codecogs.com/eqnedit.php?latex=%5Cbeta_%7BCI%7D#0) | 6.99285724437969e-06 | 1.29042055802883e-05 | 1.15130035651648e-05 | 1.63213931570087e-05 |
| [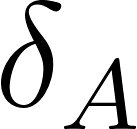](https://www.codecogs.com/eqnedit.php?latex=%5Cdelta_A#0) | 7.74757403907802e-07 | 6.13021158083943e-07 | 2.86407896814007e-07 | 5.13804618008327e-07 |
| [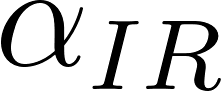](https://www.codecogs.com/eqnedit.php?latex=%5Calpha_%7BIR%7D#0) | 1.67809354564219e-09 | 1.97432760488308e-09 | 2.36370385046316e-09 | 3.45009347986944e-09 |
| [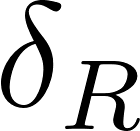](https://www.codecogs.com/eqnedit.php?latex=%5Cdelta_R#0) | 6.39077008278457e-10 | 7.54834947871794e-10 | 9.14365197748069e-10 | 1.62316970724952e-09 |
| [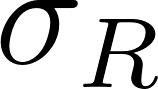](https://www.codecogs.com/eqnedit.php?latex=%5Csigma_R#0) | 2.48125353992482e-10 | 2.87539466225914e-10 | 3.81957364909534e-10 | 4.53501863133807e-10 |
| [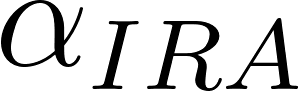](https://www.codecogs.com/eqnedit.php?latex=%5Calpha_%7BIRA%7D#0) | 1.67613785754656e-11 | 2.30383955482537e-11 | 2.45105455709727e-11 | -4.96708141102851e-12 |
| [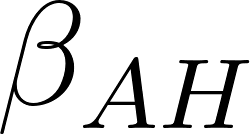](https://www.codecogs.com/eqnedit.php?latex=%5Cbeta_%7BAH%7D#0) | 9.49676416157108e-12 | 1.31637425737078e-11 | 1.15452770104941e-11 | 1.46498625425372e-11 |
| [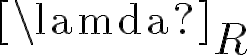](http://www.texrendr.com/?eqn=%5Clamda_R#0) | 1.31426483400791e-12 | 1.49934916190086e-12 | -8.64841299739267e-13 | 1.43370323664298e-12 |
| [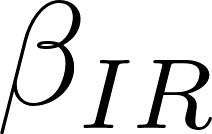](https://www.codecogs.com/eqnedit.php?latex=%5Cbeta_%7BIR%7D#0) | -1.36957825074626e-11 | -1.03124856876488e-11 | -1.05484392753183e-11 | 2.33780969293632e-11 |
| [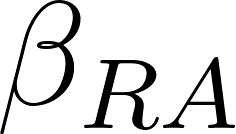](https://www.codecogs.com/eqnedit.php?latex=%5Cbeta_%7BRA%7D#0) | -3.57511687517303e-11 | -1.63550754018318e-11 | -1.27235857228927e-11 | -6.90564602297751e-11 |
| [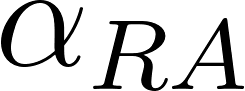](https://www.codecogs.com/eqnedit.php?latex=%5Calpha_%7BRA%7D#0) | -8.37180875151888e-11 | -9.95388276418591e-11 | -9.55244277288614e-11 | -1.26943599531086e-10 |
| [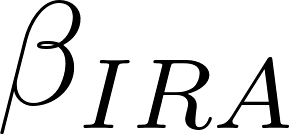](https://www.codecogs.com/eqnedit.php?latex=%5Cbeta_%7BIRA%7D#0) | -5.50406503347568e-10 | -6.46174066154559e-10 | -7.7741306088085e-10 | -9.76916213521441e-10 |
| [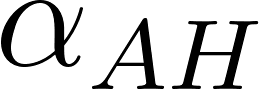](https://www.codecogs.com/eqnedit.php?latex=%5Calpha_%7BAH%7D#0) | -9.03135441489044e-10 | -2.2708702278698e-09 | -1.57356773065088e-09 | -4.19892915029736e-10 |
| [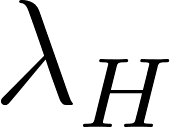](https://www.codecogs.com/eqnedit.php?latex=%5Clambda_H#0) | -2.72123944341741e-09 | -7.57346917291028e-09 | -3.7496753849853e-08 | -5.49290003467025e-08 |
| [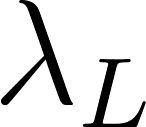](https://www.codecogs.com/eqnedit.php?latex=%5Clambda_L#0) | -1.70376187081691e-08 | -1.08555999082171e-07 | -2.26656711160892e-08 | -4.19304289210275e-09 |
| [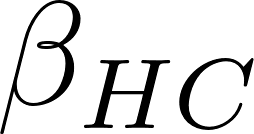](https://www.codecogs.com/eqnedit.php?latex=%5Cbeta_%7BHC%7D#0) | -2.69841149186714e-06 | -2.99695397051645e-06 | -4.32369005740768e-06 | -5.2216609315415e-06 |

**Supplementary Table 1:** This table shows the total Sobol’ sensitivity index for each parameter with a different number of base samples per column. The table is in order of descending total Sobol’ sensitivity index calculated from 175,000 base samples, which is the left-most column of index values. We began our Sobol’ sensitivity analyses with 100,000 base samples, and increased them in increments of 25,000 to study the ordering of top parameters. The 6 most-influential parameters do not change ordering between the different numbers of base samples. Other parameters do change order even between analyses with 150,000 and 175,000 base samples, so we use the ordering for the 175,000 base samples for this table and the following one

| **Supplementary Table 2: First-Order Sobol’ Sensitivity Indices, S_1_, with Different Numbers of Base Samples** | | | | |
| --- | --- | --- | --- | --- |
| **Parameter** | **Indices from 175,000 Samples** | **Indices from 150,000 Samples** | **Indices from 125,000 Samples** | **Indices from 100,000 Samples** |
| [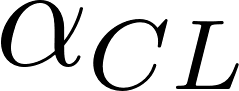](https://www.codecogs.com/eqnedit.php?latex=%5Calpha_%7BCL%7D#0) | 0.339649412078322 | 0.339925875146054 | 0.339974399357715 | 0.339318025275099 |
| [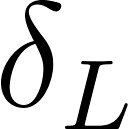](https://www.codecogs.com/eqnedit.php?latex=%5Cdelta_L#0) | 0.339216065515949 | 0.339419883383204 | 0.34005736330687 | 0.340993015342379 |
| [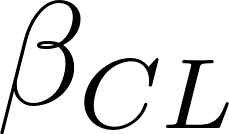](https://www.codecogs.com/eqnedit.php?latex=%5Cbeta_%7BCL%7D#0) | 0.0587726365330789 | 0.0588677584366251 | 0.0590161518536422 | 0.0589545448508023 |
| [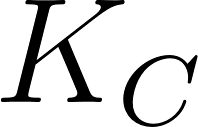](https://www.codecogs.com/eqnedit.php?latex=K_C#0) | 0.0582327433010136 | 0.0581947464230351 | 0.0582825391428798 | 0.0582618791834558 |
| [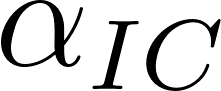](https://www.codecogs.com/eqnedit.php?latex=%5Calpha_%7BIC%7D#0) | 0.0260502814257566 | 0.0260315169834607 | 0.0260626715461808 | 0.0262484418589166 |
| [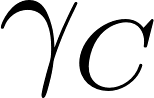](https://www.codecogs.com/eqnedit.php?latex=%5Cgamma_C#0) | 0.0243516126700855 | 0.0246858779098501 | 0.0248640686176368 | 0.0244104165566139 |
| [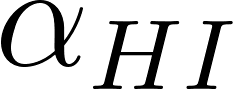](https://www.codecogs.com/eqnedit.php?latex=%5Calpha_%7BHI%7D#0) | 0.01146760643643 | 0.0115113036093388 | 0.0110409044412268 | 0.0107342076530564 |
| [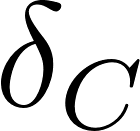](https://www.codecogs.com/eqnedit.php?latex=%5Cdelta_C#0) | 0.0173613480698686 | 0.0175151898602036 | 0.0171610003653279 | 0.0171853386678728 |
| [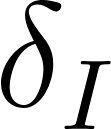](https://www.codecogs.com/eqnedit.php?latex=%5Cdelta_I#0) | 0.0084887010908517 | 0.00828390577669817 | 0.00850618955877249 | 0.0082590698484659 |
| [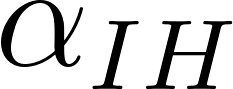](https://www.codecogs.com/eqnedit.php?latex=%5Calpha_%7BIH%7D#0) | 0.00354268561934134 | 0.00365474094088385 | 0.00358775213880826 | 0.00384903310108231 |
| [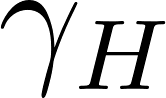](https://www.codecogs.com/eqnedit.php?latex=%5Cgamma_H#0) | 0.00355605715508874 | 0.00348311966687893 | 0.00297571453196656 | 0.00332967581402795 |
| [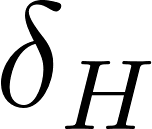](https://www.codecogs.com/eqnedit.php?latex=%5Cdelta_H#0) | 0.00211417200631198 | 0.00201857941026906 | 0.00200272592620782 | 0.00144081097254787 |
| [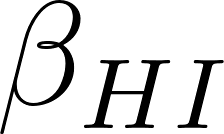](https://www.codecogs.com/eqnedit.php?latex=%5Cbeta_%7BHI%7D#0) | 0.00200070655825804 | 0.00179378535843735 | 0.0014798523618249 | 0.00138537101507352 |
| [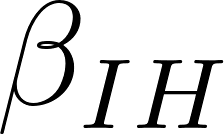](https://www.codecogs.com/eqnedit.php?latex=%5Cbeta_%7BIH%7D#0) | 0.00095279641696444 | 0.000852549927781191 | 0.000724598372773306 | 0.000255060995570715 |
| [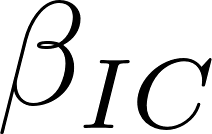](https://www.codecogs.com/eqnedit.php?latex=%5Cbeta_%7BIC%7D#0) | 0.00437707290630445 | 0.00450772599968428 | 0.00444952863413971 | 0.00445242330397608 |
| [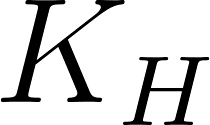](https://www.codecogs.com/eqnedit.php?latex=K_H#0) | 0.00100625125307626 | 0.00113094340676358 | 0.0010267023842836 | 0.00107797224221765 |
| [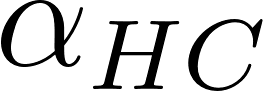](https://www.codecogs.com/eqnedit.php?latex=%5Calpha_%7BHC%7D#0) | -3.3082119922446e-05 | -2.23224198062005e-05 | -2.5096836905465e-05 | -2.09389018732511e-05 |
| [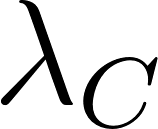](https://www.codecogs.com/eqnedit.php?latex=%5Clambda_C#0) | 3.06286081504965e-06 | 8.13434807795762e-07 | 3.62783673380463e-06 | 6.8411111284052e-06 |
| [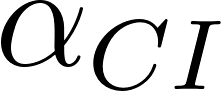](https://www.codecogs.com/eqnedit.php?latex=%5Calpha_%7BCI%7D#0) | 8.61775287119046e-06 | 8.89194956667903e-06 | 4.48347702212346e-06 | 3.05041972659571e-06 |
| [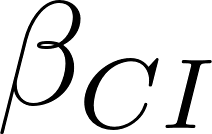](https://www.codecogs.com/eqnedit.php?latex=%5Cbeta_%7BCI%7D#0) | -3.18282904353376e-06 | -5.22857253985394e-06 | -8.67080254171133e-06 | -1.11161195871706e-05 |
| [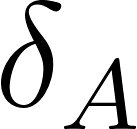](https://www.codecogs.com/eqnedit.php?latex=%5Cdelta_A#0) | 1.17033148092505e-06 | 1.11717434639924e-06 | 1.14512127396597e-06 | 1.11943859477737e-06 |
| [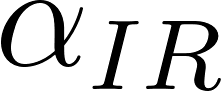](https://www.codecogs.com/eqnedit.php?latex=%5Calpha_%7BIR%7D#0) | -5.19688479219526e-10 | -6.14797340477186e-10 | -7.45992291070277e-10 | -6.6553189695691e-10 |
| [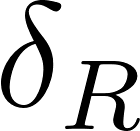](https://www.codecogs.com/eqnedit.php?latex=%5Cdelta_R#0) | 5.40929304774742e-10 | 5.06058078465932e-10 | 6.1384661552835e-10 | 1.03769119146481e-09 |
| [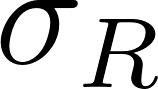](https://www.codecogs.com/eqnedit.php?latex=%5Csigma_R#0) | -1.15480087530314e-10 | -1.27777459500361e-10 | -1.494786023142e-10 | -1.73227754031926e-10 |
| [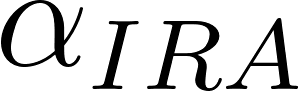](https://www.codecogs.com/eqnedit.php?latex=%5Calpha_%7BIRA%7D#0) | 4.53598335998037e-12 | 1.31450751304765e-12 | -1.03569954996998e-11 | -5.73462899686239e-13 |
| [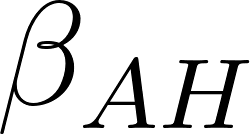](https://www.codecogs.com/eqnedit.php?latex=%5Cbeta_%7BAH%7D#0) | 6.15563798773305e-12 | 5.82028314582943e-12 | 7.6306791305525e-12 | 1.57469961615668e-11 |
| [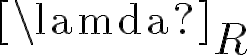](http://www.texrendr.com/?eqn=%5Clamda_R#0) | -4.50277958278915e-13 | -1.88566976709298e-12 | -8.42591237371402e-13 | -1.40352468676912e-12 |
| [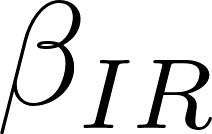](https://www.codecogs.com/eqnedit.php?latex=%5Cbeta_%7BIR%7D#0) | 3.52696904222354e-11 | 4.45611464044156e-12 | 3.47477518524923e-12 | 2.6588950465265e-11 |
| [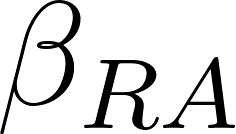](https://www.codecogs.com/eqnedit.php?latex=%5Cbeta_%7BRA%7D#0) | 4.06451841171458e-11 | 4.19397797247032e-11 | -3.36108398549562e-11 | 4.7799308413387e-11 |
| [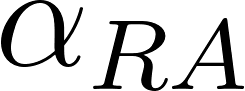](https://www.codecogs.com/eqnedit.php?latex=%5Calpha_%7BRA%7D#0) | 9.08302652119633e-11 | 6.12842102422036e-11 | 1.74053991631181e-10 | 1.60968133819534e-10 |
| [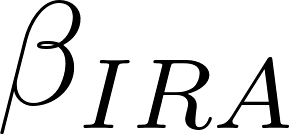](https://www.codecogs.com/eqnedit.php?latex=%5Cbeta_%7BIRA%7D#0) | -4.95874344548754e-10 | -5.8369698241944e-10 | -6.78070897367758e-10 | -8.50598100238736e-10 |
| [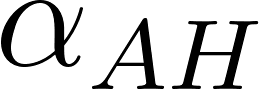](https://www.codecogs.com/eqnedit.php?latex=%5Calpha_%7BAH%7D#0) | -2.64414277841109e-09 | -2.919831664628e-09 | -2.00407830701432e-09 | -1.37258158497639e-09 |
| [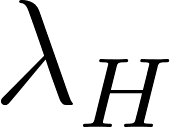](https://www.codecogs.com/eqnedit.php?latex=%5Clambda_H#0) | -4.84317089454712e-08 | -5.26682113436572e-08 | -6.76127638018383e-08 | -8.62359112446512e-08 |
| [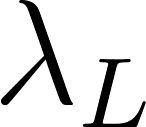](https://www.codecogs.com/eqnedit.php?latex=%5Clambda_L#0) | -1.84973703824753e-08 | -5.08127998502795e-08 | -5.5638685709603e-09 | -3.04394547978836e-08 |
| [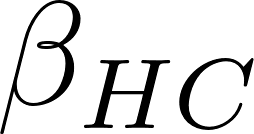](https://www.codecogs.com/eqnedit.php?latex=%5Cbeta_%7BHC%7D#0) | 2.95039150526057e-06 | 2.93229609327302e-06 | 2.77307435887906e-06 | 3.77811156115105e-06 |

**Supplementary Table 2:** This table shows the first-order Sobol’ sensitivity index for each parameter with a different number of base samples per column. The ordering in this table follows that of **Supplementary Table 1**, which is ordered by total Sobol’ sensitivity index values


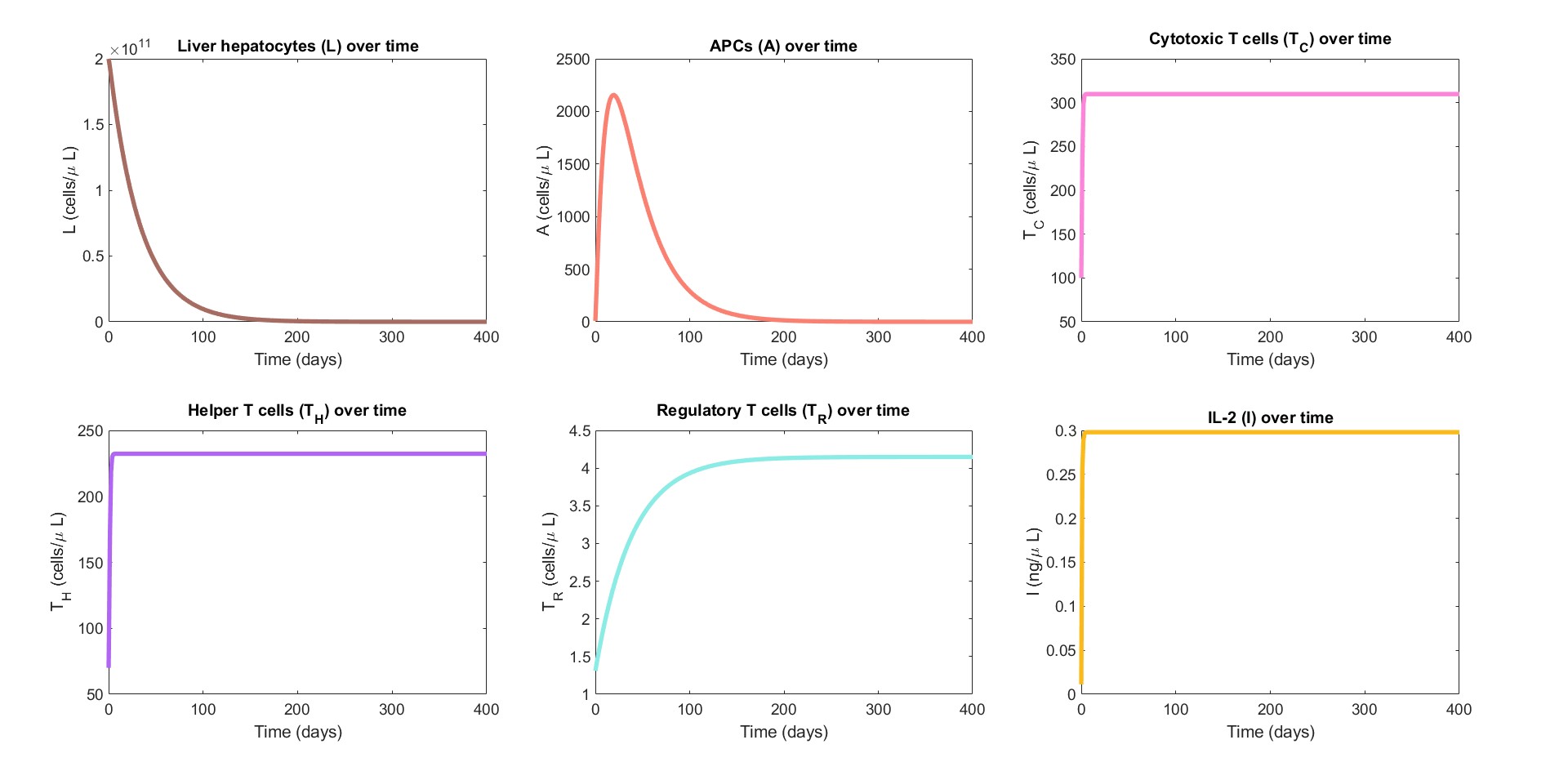


**Fig. 1** Simulation of model variables for 400 days, in order to study theoretical steady-state values if treatment continued to be withheld


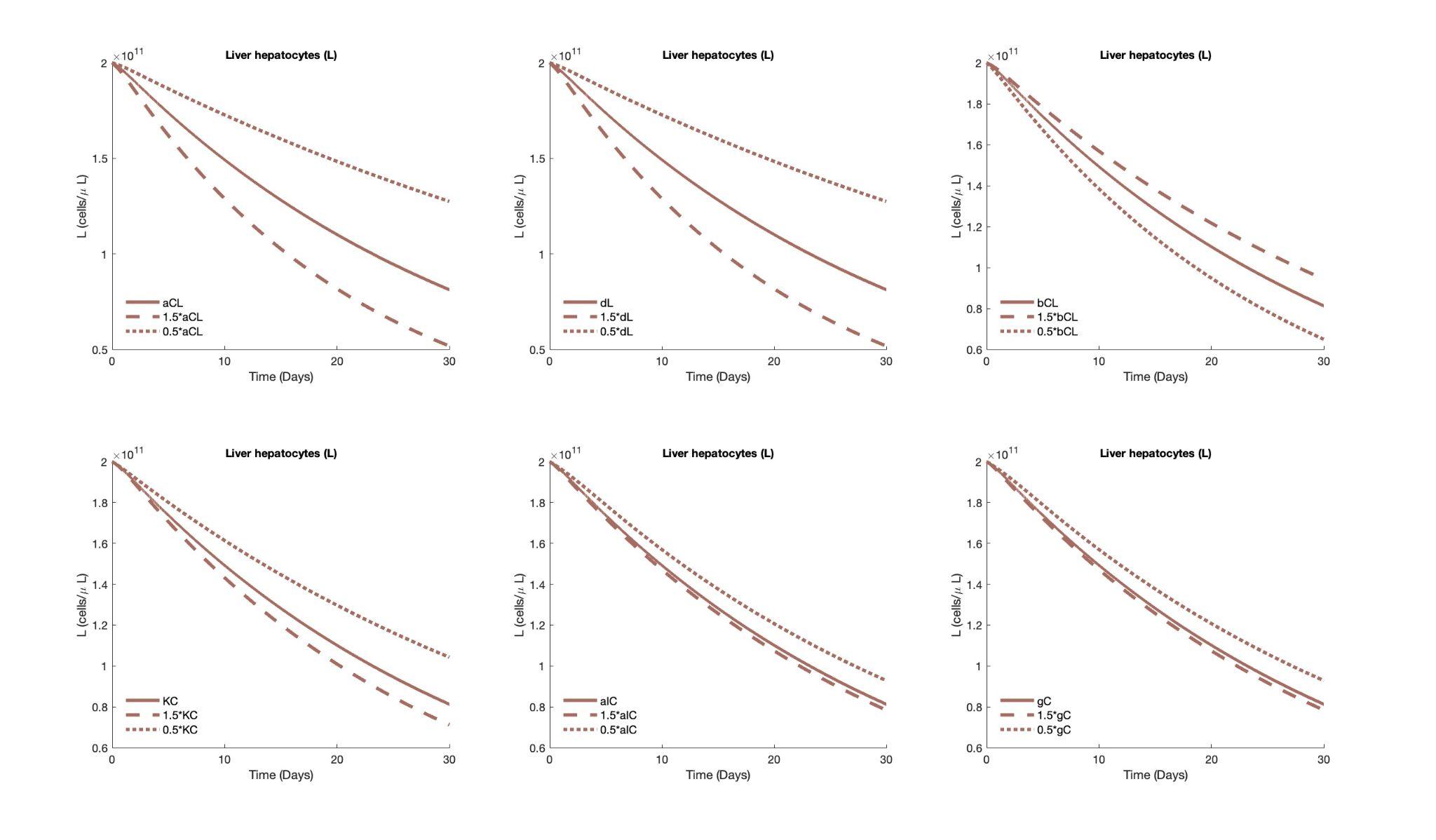


**Fig. 2a** Effect of parameter changes on healthy liver hepatocyte levels. Solid curves show hepatocyte levels (L) with nominal original parameter values. Effect of one-at-a-time changes in each of the top 6 most-influential parameters. Dashed curves are simulations of L with the parameter 50% higher than its nominal value. Dotted curves are simulations of L with the parameter 50% lower than its nominal value


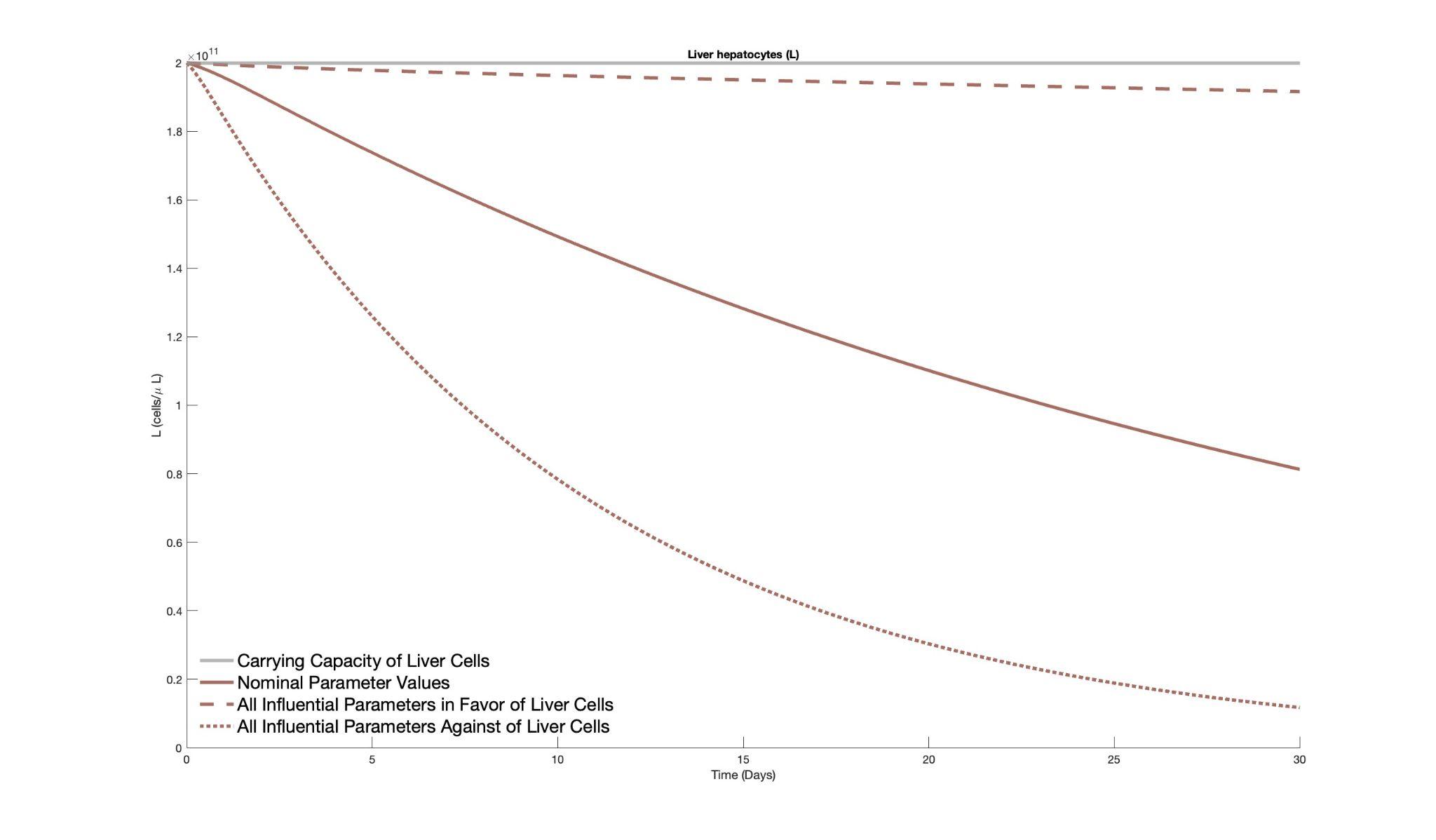


**Fig. 2b** Effect of parameter changes on healthy liver hepatocyte levels. Simulation of L when all changes are in favor (dashed) or all are against (dotted). Specifically, the dashed curve shows L when [
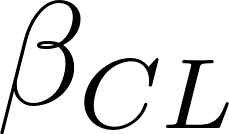
](https://www.codecogs.com/eqnedit.php?latex=%5Cbeta_%7BCL%7D#0) was increased and the other 5 of the 6 most-influential parameters were decreased. The dotted curve shows L with the reverse: [
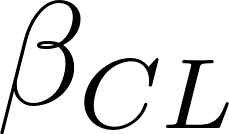
](https://www.codecogs.com/eqnedit.php?latex=%5Cbeta_%7BCL%7D#0) was decreased and the other 5 most-influential parameters were increased. The solid gray horizontal line shows the carrying capacity of liver cells
